# The translation initiating factor eIF4E and arginine methylation underlie G3BP1 function in dendritic spine development of neurons

**DOI:** 10.1016/j.jbc.2023.105029

**Published:** 2023-07-11

**Authors:** Rui Dong, Xuejun Li, Angelo D. Flores, Kwok-On Lai

**Affiliations:** 1 Department of Neuroscience, City University of Hong Kong, Hong Kong, China; 2 Hong Kong Institute for Advanced Study, City University of Hong Kong, Hong Kong, China

**Keywords:** dendritic spine, synapse, arginine methylation, RNA-binding protein, signal transduction

## Abstract

Communication between neurons relies on neurotransmission that takes place at synapses. Excitatory synapses are located primarily on dendritic spines that possess diverse morphologies, ranging from elongated filopodia to mushroom-shaped spines. Failure in the proper development of dendritic spines has detrimental consequences on neuronal connectivity, but the molecular mechanism that controls the balance of filopodia and mushroom spines is not well understood. G3BP1 is the key RNA-binding protein that assembles the stress granules in non-neuronal cells to adjust protein synthesis upon exogenous stress. Emerging evidence suggests that the biological significance of G3BP1 extends beyond its role in stress response, especially in the nervous system. However, the mechanism underlying the regulation and function of G3BP1 in neurons remains elusive. Here we found that G3BP1 suppresses protein synthesis and binds to the translation initiation factor eIF4E *via* its NTF2-like domain. Notably, the over-production of filopodia caused by G3BP1 depletion can be alleviated by blocking the formation of the translation initiation complex. We further found that the interaction of G3BP1 with eIF4E is regulated by arginine methylation. Knockdown of the protein arginine methyltransferase PRMT8 leads to elevated protein synthesis and filopodia production, which is reversed by the expression of methylation-mimetic G3BP1. Our study, therefore, reveals arginine methylation as a key regulatory mechanism of G3BP1 during dendritic spine morphogenesis and identifies eIF4E as a novel downstream target of G3BP1 in neuronal development independent of stress response.

Excitatory neurotransmission occurs on the postsynaptic neuron at dendritic spines that exist as diverse morphologies. The mushroom-shaped spines with large heads and distinct spine necks are the mature spines for memory storage (1), but there are also elongated filopodia that have a molecular composition distinct from mushroom spines (2). Filopodia represent the spine precursors during synaptogenesis (3, 4), while in the adult brain, they undergo rapid turnover (5) and may be involved in fast learning (6). As the brain matures, there is an increasing number of mushroom spines and a reduction of filopodia in neurons. This developmental switch of spine morphology is essential for brain function, as exemplified in Fragile-X syndrome and autism spectrum disorders, in which the prevalence of thin spines and filopodia is associated with various cognitive deficits (7, 8). It is therefore important to elucidate the molecular mechanism that suppresses the over-production of filopodia during dendritic spine maturation.

The proper development and function of synapses require tight regulation of neuronal protein synthesis. One crucial control point occurs at the level of translation initiation complex formation, which involves the interaction of the translation initiation factors eIF4E and eIF4G with the 5′-capped mRNAs and is regulated by mTOR-mediated phosphorylation of 4EBP-1 (9). Disruption of the mTOR signaling such as the phosphorylation of its downstream target S6 kinase inhibits dendritic spine formation (10). On the other hand, uncontrolled mTOR signaling and the subsequent elevation in protein synthesis are associated with synaptic and behavioral defects in autism (11, 12). In mice lacking the RNA-binding protein (RBP) FMRP, which carries mRNAs to dendrites and represses their local protein synthesis (13, 14), the spine defects and behavioral abnormalities can be alleviated by reducing the eIF4E-eIF4G interaction (15). Besides FMRP, depletion of some other dendritically localized RBPs, such as Staufen and FUS, also increases the formation of elongated spines (16, 17). It is currently unclear whether the other dendritic RBPs also act on the translation initiation complex to promote dendritic spine development.

Many RBPs contain the glycine-arginine-rich (RGG) motif which is a hot spot for arginine methylation (18), a major form of protein post-translational modification in the nucleus where it is critically involved in chromatin remodeling, gene transcription, and RNA splicing (19, 20). Recent proteomic studies have identified many putative arginine-methylated proteins in the brain, which surprisingly include pre- and postsynaptic proteins (21). Nonetheless, the role of arginine methylation in synapse development and function is not well-defined. Arginine methylation is catalyzed by the enzyme protein arginine methyltransferases (PRMTs). Among the nine mammalian PRMTs (19), PRMT8 is particularly interesting because it is the only membrane-bound PRMT and its expression is restricted in the brain (22, 23). Knockout mice lacking PRMT8 display impaired development of dendrites and synapses as well as memory deficits (24, 25, 26). PRMT8 is also present in dendritic spines and it promotes dendritic spine maturation through regulation of the Rac-PAK signaling and actin dynamics (27).

Ras-GTPase activating protein SH3 domain binding proteins (G3BPs) are proteins pulled down by PRMT8 from the mouse brain (27). G3BPs are two homologous RBPs, namely, G3BP1 and G3BP2, that are encoded by two distinct genes (28). G3BP1 is one of the key proteins that assembles stress granules as a cellular response to different exogenous stresses, such as oxidative stress, nutrient deprivation, and viral infection (29, 30, 31). These transient stress granules arrest the translation of most mRNAs and help the cell survive (32). Altered G3BP1 expression has been linked to cancer, neurodegeneration, and nerve injury (33, 34, 35, 36, 37), underscoring the importance of G3BP1 and stress granules in the control of cellular proteostasis under adverse conditions. However, increasing evidence suggests that G3BP1 also has important physiological functions in the absence of stress stimulus, especially in the nervous system (38). G3BP1 is present in dendrites and dendritic spines, and its depletion causes defective actin dynamics as well as the over-production of filopodia (27). High-throughput sequencing reveals numerous transcripts that may interact with G3BP1 in the mouse brain, with an over-representation of transcripts involved in synaptic transmission (39). G3BP1 knockout hippocampal neurons also exhibit exaggerated protein synthesis-dependent long-term depression (34), which is reminiscent of that of FMRP knockout neurons. Nonetheless, much of our understanding of the regulation, function, and mechanism of G3BP1 is derived from stress granules in non-neuronal cells. How does G3BP1 regulate neuronal function in a physiological context without stress is still unclear. Moreover, whether G3BP1 is the key downstream target to mediate PRMT8 function in neurons has not been addressed. Given that the protein interaction network of G3BP1 in neurons is distinct from that in non-neuronal cells (40), it is important to identify and characterize the downstream targets of G3BP1 in neurons as well as to elucidate the role of methylation in regulating its function under normal physiological condition. In this study, we reveal the translation initiation factor eIF4E and the importance of arginine methylation within the RGG motif in mediating G3BP1 function in dendritic spine maturation of neurons.

## Results

### G3BP1 interacts with the translation initiation factor eIF4E and suppresses mRNA translation

**Figure 1:**
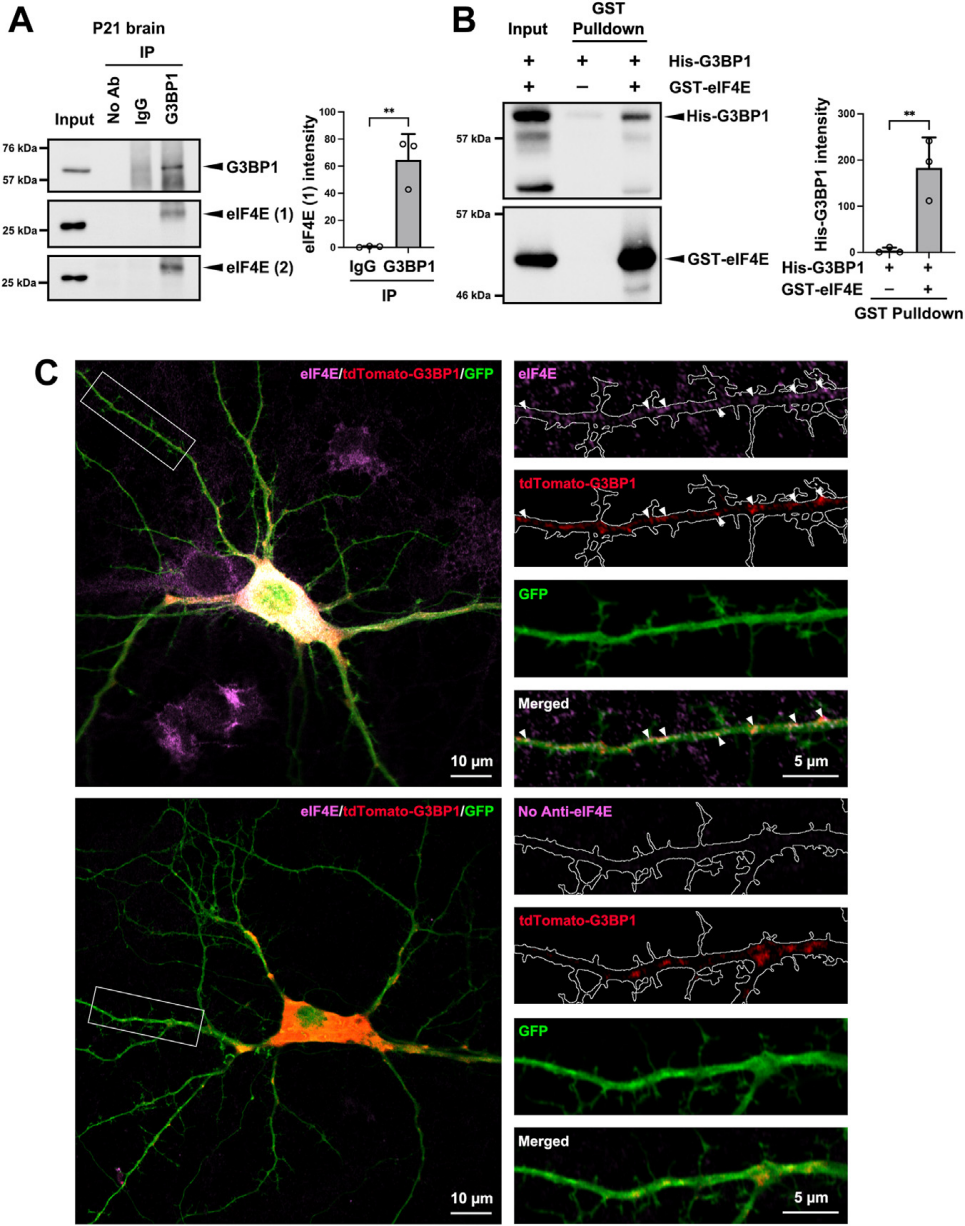
**G3BP1 interacts with the translation initiation factor eIF4E.***A*, eIF4E was co-immunoprecipitated with G3BP1 in the mouse brain at postnatal day 21 (P21). eIF4E (1) and eIF4E (2) represented Western blots with two different antibodies that recognize distinct regions of eIF4E (see Experimental procedures). Representative Western blots were shown. Three independent experiments were performed. Data were mean ± SD; ∗∗*p* < 0.01; Student’s *t* test. *B*, direct binding between G3BP1 and eIF4E *in vitro*. Recombinant purified His-G3BP1 protein was pulled down by GST-eIF4E in cell-free conditions. Representative Western blots with G3BP1 (*upper*) and eIF4E (*lower*) antibodies were shown. Three independent experiments were performed. Data were mean ± SD; ∗∗*p* < 0.01; Student’s *t* test. *C*, representative images showing the colocalization of eIF4E (*magenta*) and tdTomato-G3BP1 (*red*) in the dendrite. Hippocampal neurons were co-transfected with tdTomato-G3BP1 and GFP at 12 DIV, followed by staining of eIF4E and GFP at 16 DIV. Overlap puncta of eIF4E and Tdtomato-G3BP1 were indicated by arrowheads (14 dendrites from 14 neurons in one experiment were analyzed, Pearson’s Coefficient 0.769). The absence of an eIF4E antibody (*lower panel*) served as the negative control to rule out crosstalk between the two fluorescence channels. The eIF4E staining outside the transfected neurons was attributed to the neighboring non-transfected neurons, which expressed the endogenous eIF4E but without GFP and Tdtomato-G3BP1.

When the translation is stalled upon the presence of cellular stress, various translation initiation factors that comprise the noncanonical 48S preinitiation complex are present in stress granules (32). We, therefore, ask whether translation initiation factors and the regulation of protein synthesis might mediate the function of G3BP1 in neurons without exogenous stress. Consistent with this notion, we found that the translation initiation factor eIF4E was co-immunoprecipitated with G3BP1 in the mouse brain (Fig. 1*A*). Similar co-immunoprecipitation of the two proteins was observed using another eIF4E antibody that recognized a different epitope (Fig. 1*A*). *In vitro* pull-down experiment was further performed using purified recombinant GST-eIF4E and His-G3BP1, which indicated that the two recombinant proteins could bind to each other directly in cell-free conditions (Fig. 1*B*). eIF4E is present near synapses and is implicated in local translation of dendritic mRNAs (41). We, therefore, determine whether G3BP1 is co-localized with eIF4E in dendrites. After co-transfecting cultured hippocampal neurons with the GFP plasmid and a construct of G3BP1 tagged with tdTomato, we performed immunofluorescence staining with anti-eIF4E antibody, whose specificity in immunohistochemistry has been previously demonstrated (42, 43). We confirmed its staining specificity in hippocampal neurons by transfecting the neurons with eIF4E shRNA, which significantly reduced the staining intensity by the eIF4E antibody when compared to a control shRNA (Fig. S1). Upon immunofluorescence staining, we observed discrete small aggregates of tdTomato-G3BP1 on the GFP-labeled dendrites, where they over-lapped substantially with endogenous eIF4E (Fig. 1*C*) (43.8% eIF4E puncta overlapped with tdTomato-G3BP1 and 44.9% tdTomato-G3BP1 puncta overlapped with eIF4E; 14 dendrites were analyzed, Pearson’s Coefficient 0.769). These findings suggest that the translation initiation factor eIF4E could be a downstream effector to mediate G3BP1 function in neuronal dendrites under normal physiological conditions independent of stress granule formation.

**Figure 2:**
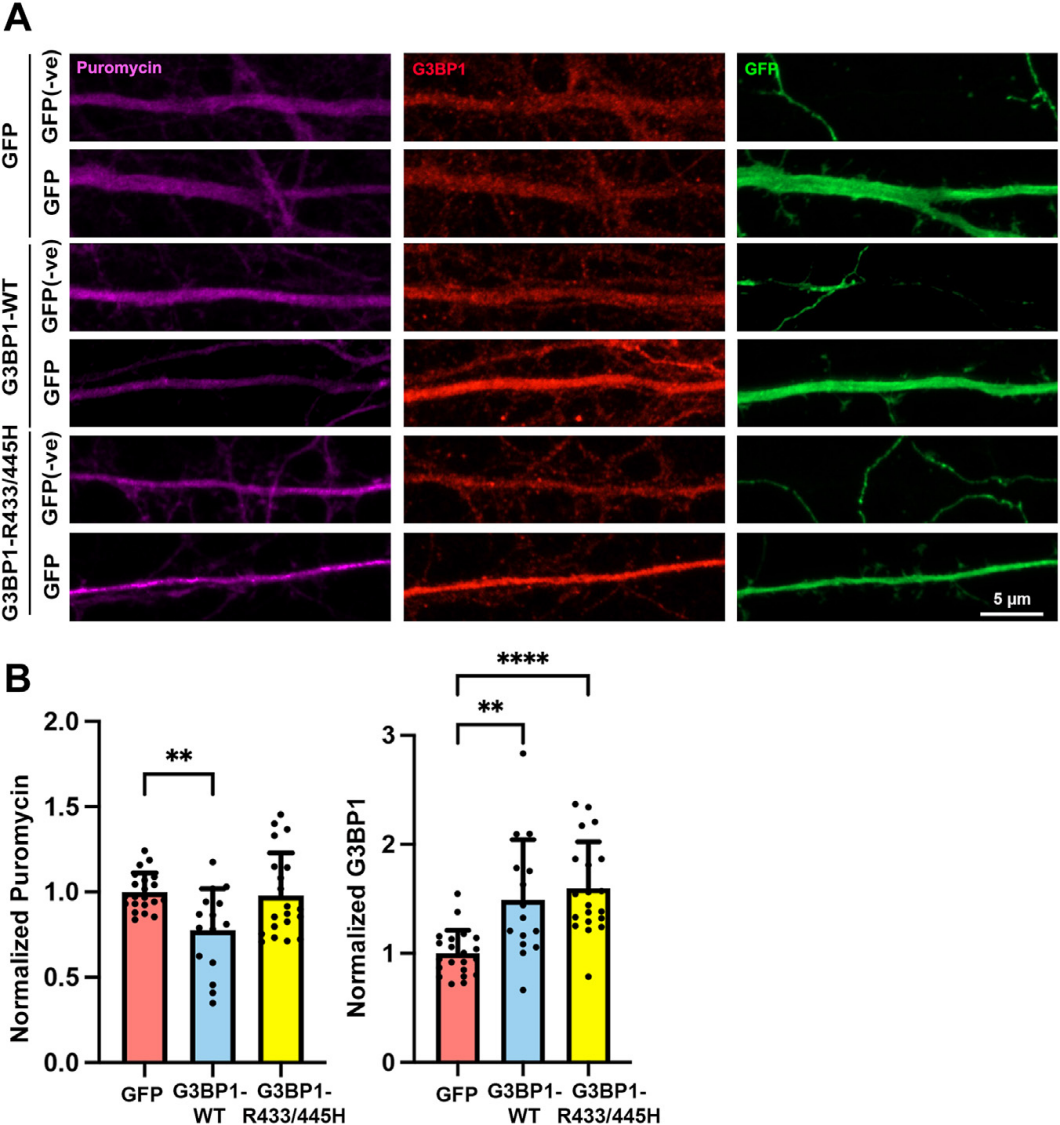
**G3BP1 suppresses protein synthesis in hippocampal neurons.***A*, hippocampal neurons were transfected with GFP, or GFP together with wild-type (WT) G3BP or methylation-deficient G3BP1 (R433/445H) at 13 DIV, followed by puromycin (*magenta*) labeling for 10 min at 16 DIV. Neurons were stained with G3BP1 (*red*), puromycin (*magenta*), and GFP (*green*). Representative images were shown. GFP(-ve) represented dendrites without GFP signal from neighboring non-transfected control neurons. *B*, normalized puromycin or G3BP1 was calculated as the ratio of fluorescence intensity in the dendrites from transfected (GFP-positive, “GFP”) neurons to the neighboring non-transfected (GFP-negative, “GFP -ve”) neurons. The ratio of the GFP control group was set as “1”. Results were pooled from three independent experiments; 15 to 21 dendrites from 15 to 21 neurons were quantified for each condition. Data were mean ± SD; ∗∗*p* < 0.01, ∗∗∗∗*p* < 0.0001; one-way ANOVA, Tukey’s multiple comparison test in the puromycin quantification; Kruskal-Wallis test, Dunn’s multiple comparison test in the G3BP1 quantification.

To ask whether G3BP1 regulates neuronal mRNA translation in dendrites, we performed the surface sensing of translation (SUnSET) assay based on the aminoacylated tRNA analog puromycin (44). After adding to the medium, puromycin is incorporated into the newly synthesized proteins and results in the premature termination of translation. The newly synthesized proteins can then be detected *in situ* using the puromycin antibody and immunofluorescence microscopy. The high sensitivity, ease of use, and quick labeling make SUnSET a good method to detect the location of proteins undergoing active synthesis. After 10-min incubation, puromycin labeling was detected in both the neuronal cell body and on dendrites of cultured hippocampal neurons. No labeling was observed either in the absence of puromycin or without anti-puromycin antibody. Furthermore, the puromycin signals were largely reduced when neurons were pre-treated with the protein synthesis inhibitor anisomycin, indicating the specificity of puromycin in labeling the newly synthesized proteins (Fig. S2). Measuring puromycin intensity would therefore enable us to determine the role of G3BP1 on mRNA translation. Toward this end, we co-transfected hippocampal neurons with GFP and an expression construct of G3BP1. The puromycin and G3BP1 staining intensities were then compared between the dendrites of transfected (GFP-positive, “GFP”) neurons and the nearby non-transfected (GFP-negative, “GFP-ve”) neurons within the same imaging fields. In the transfection, the co-transfected plasmid was in 4-fold excess than that of GFP, which makes GFP a reliable indicator for the presence of the other co-transfected plasmid (Fig. S3). Over-expression of G3BP1 resulted in lower puromycin intensity on the dendrites than those from nearby non-transfected neurons (Fig. 2, *A* and *B*). The decrease in puromycin intensity was not an artifact of over-expressing an exogenous protein because a similar reduction of puromycin staining was not observed in the negative control in which the same amount of GFP plasmid was transfected. G3BP1 undergoes arginine methylation at R433 and R445 located within the C-terminal RGG motif (21, 45). The methylation-deficient G3BP1 (with the substitution of arginine to histidine) fails to promote dendritic spine maturation (27). Consistent with the hypothesis that suppression of protein synthesis underlies G3BP1 function in spine morphogenesis, only the over-expression of wild-type but not the methylation-deficient (R433/445H) G3BP1 significantly reduced the puromycin intensity on dendrites in the SUnSET assay (Fig. 2, *A* and *B*). Taken together, these findings indicate that G3BP1 suppresses mRNA translation in neurons, and it interacts with the translation initiation factor eIF4E.

### G3BP1 promotes dendritic spine maturation *via* NTF2-like domain and eIF4E

**Figure 3:**
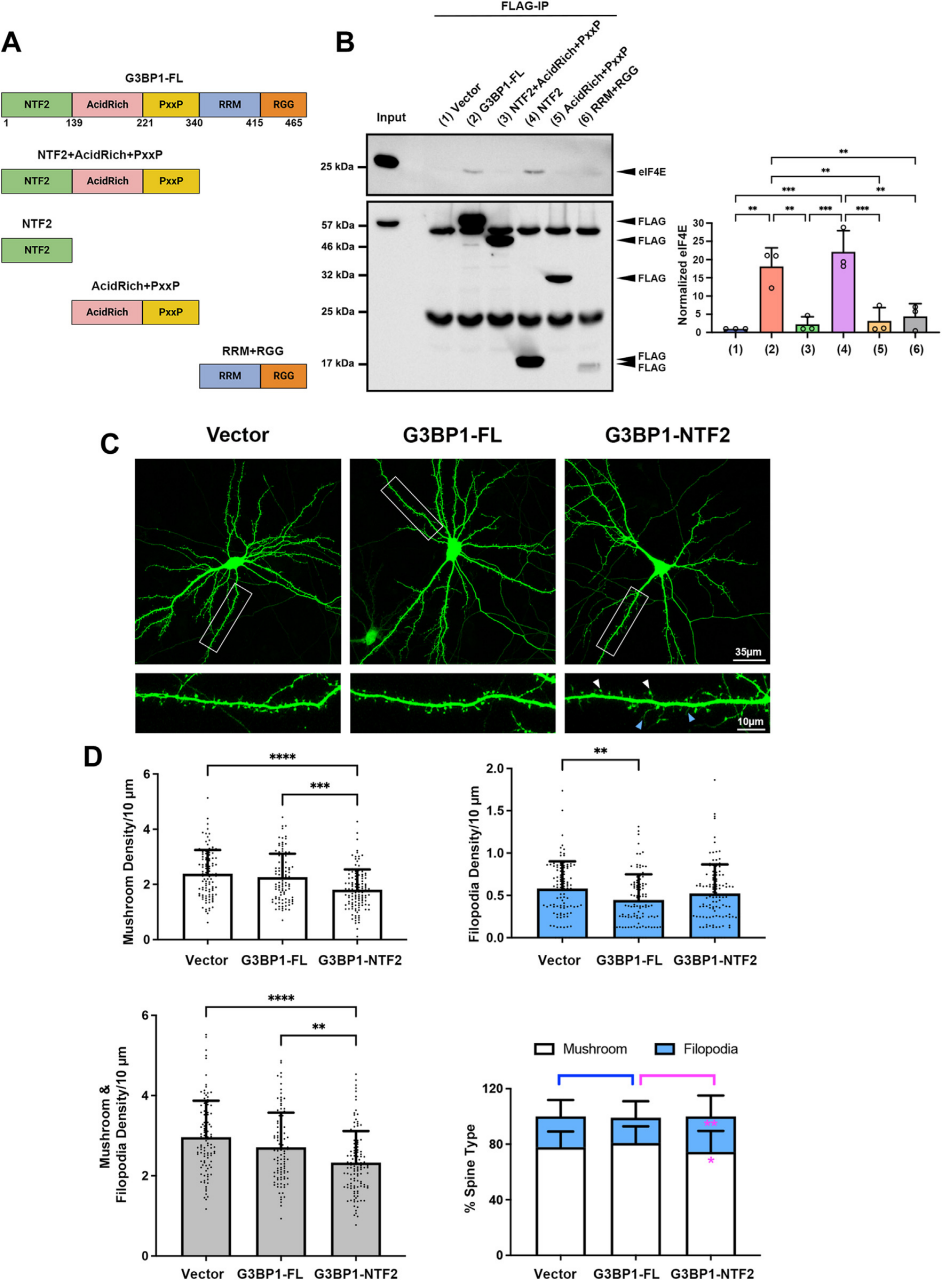
**G3BP1 interacts with eIF4E *via* the NTF2-like domain and this interaction regulates the formation of mushroom spines.***A*, schematic diagram illustrating the various domains of G3BP1. The numbers denote the amino acid sequence corresponding to rat G3BP1. *B*, the different FLAG-tagged G3BP1 constructs were transfected into HEK293T cells and immunoprecipitated by FLAG beads. Endogenous eIF4E was pulled down by full-length (G3BP1-FL) and NTF2-like domain (NTF2) of G3BP1 but not the other three truncated G3BP1 proteins (*top*). Expression of the different FLAG-tagged constructs was indicated by Western blot with anti-FLAG antibody (*bottom*). The two bands near 50 kDa and 23 kDa represented the heavy chain and light chain of the FLAG antibody in the beads. Results were pooled from three independent experiments. Data were mean ± SD; ∗∗*p* < 0.01, ∗∗∗*p* < 0.001; One-way ANOVA, Tukey’s multiple comparisons test. *C*, expression of the NTF2-like domain of G3BP1 disrupted the formation of mushroom spines. Cultured hippocampal neurons (13 DIV) were co-transfected with GFP and pcDNA3 (vector), full-length G3BP1 (G3BP1-FL) or the NTF2-like domain of G3BP1 (G3BP1-NTF2). Representative images were shown. Mushroom spines and filopodia were indicated by *white* and *blue arrowheads*, respectively. *D*, quantification indicated that the exogenous expression of NTF2-like domain (NTF2), but not full-length G3BP1 (G3BP1-FL), reduced mushroom spine density. In contrast, filipodia density was reduced by G3BP1-FL but remained unchanged by NTF2 expression. Results were pooled from three independent experiments; 104 to 114 dendrites from 36 to 41 neurons were quantified for each condition. Data were mean ± SD; ∗*p* < 0.05, ∗∗*p* < 0.01, ∗∗∗*p* < 0.001, ∗∗∗∗*p* < 0.0001; Kruskal-Wallis test, Dunn’s multiple comparison test.

G3BP1 is composed of several distinct domains: the N-terminal nuclear transport factor 2 (NTF2)-like domain, the central acid-rich and proline-rich (PxxP) motifs, and the C-terminal RNA-binding domain consisting of the RNA recognition motif (RRM) and the arginine-glycine rich (RGG) motif (Fig. 3*A*). To identify which domain of G3BP1 binds to eIF4E, we generated FLAG-tagged expression constructs with different combinations of G3BP1 domains, followed by an expression in 293T cells and co-immunoprecipitation by FLAG beads to detect eIF4E. The NTF2-like domain but not the other domains was sufficient for the interaction with eIF4E that was comparable to full-length G3BP1 (Fig. 3*B*). Surprisingly, deletion of the RNA-binding domain renders the remaining G3BP1 (NTF2 + AcidRich + PxxP) unable to interact with eIF4E, although the NTF2-like domain is still present. Therefore, G3BP1 utilizes its NTF2-like domain to bind to eIF4E, but its RNA-binding domain is permissive for this interaction with full-length G3BP1. It is unclear whether the RNA-binding domain by itself can also interact with eIF4E since its expression in multiple experiments was consistently much lower than the other domains of G3BP1 (Fig. 3*B*).

To ask whether the interaction between G3BP1 and eIF4E is functionally important, we transfected hippocampal neurons with the full-length or NTF2-like domain of G3BP1 and examined the consequences on dendritic spine morphogenesis. We reason that the over-expression of the NTF2-like domain will interfere with the interaction between endogenous eIF4E and G3BP1 and hence inhibit their functions in a dominant-negative manner. Indeed, compared to vector control, over-expressing the NTF2-like domain of G3BP1 significantly reduced the density of mushroom spines while the number of filopodia remained unchanged. In contrast, over-expressing the full-length G3BP1 (G3BP1-FL) did not disrupt the mushroom spines. Instead, the filopodia density was significantly reduced (Fig. 3, *C* and *D*).

**Figure 4:**
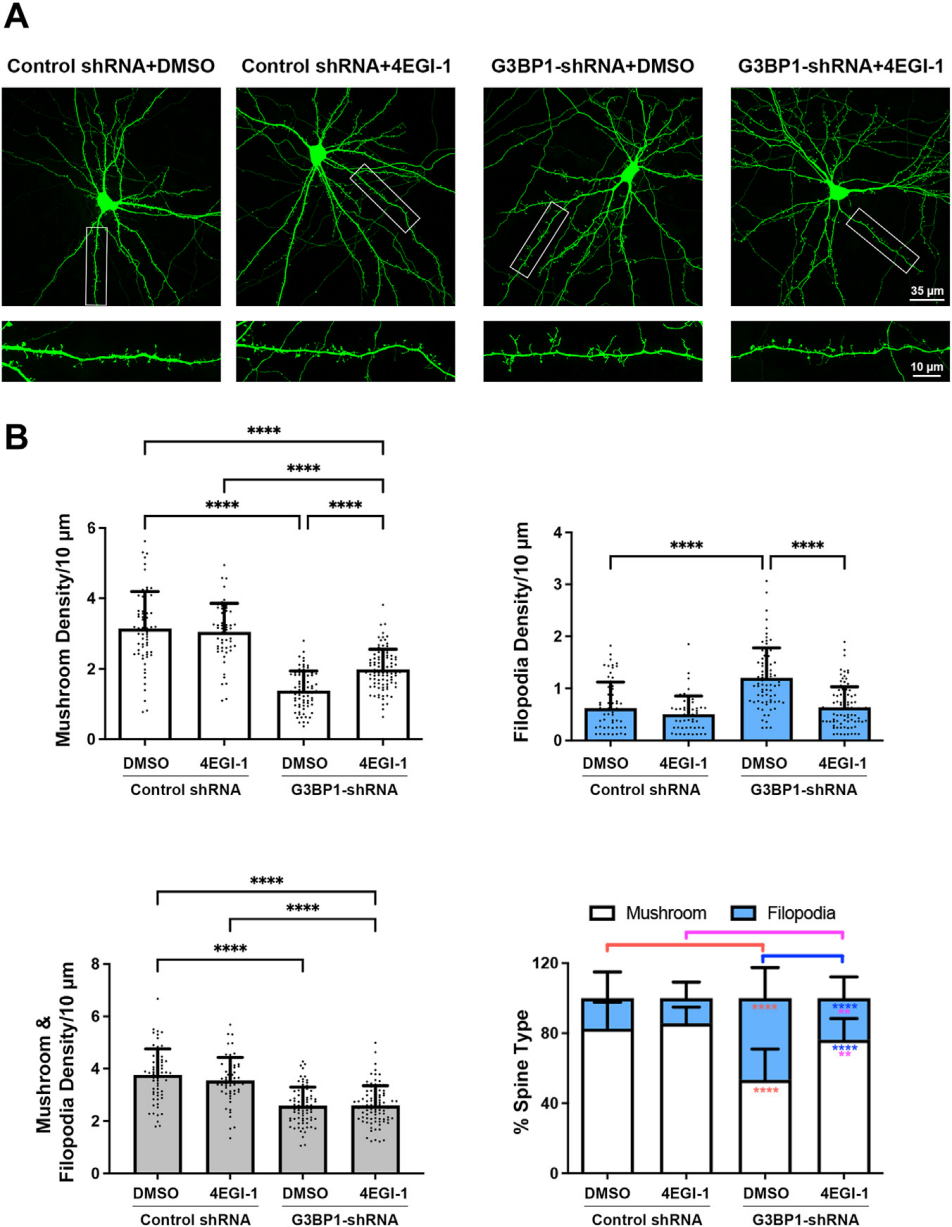
**G3BP1 regulates dendritic spine maturation *via* eIF4E.***A*, cultured hippocampal neurons (13 DIV) were transfected with GFP, control-shRNA or G3BP1-shRNA, followed by treatment with DMSO (as vehicle control) or the eIF4E inhibitor 4EGI-1 (50 μM) for 4 h at 16 DIV. Representative images were shown. *B*, quantification indicated that the knockdown of G3BP1 significantly reduced the mushroom spines and increased filopodia density in DMSO, but the presence of 4EGI-1 abolished the elevated filopodia density caused by the G3BP1 knockdown and partially restored the mushroom spine formation. Results were pooled from two independent experiments; 55 to 95 dendrites from 25 to 33 neurons were quantified for each condition. Data were mean ± SD; ∗∗*p* < 0.01, ∗∗∗∗*p* < 0.0001; one-way ANOVA, Tukey’s multiple comparison test (mushroom spine density and mushroom and filopodia density) or Kruskal-Wallis test, Dunn’s multiple comparison test (filopodia density and the percentage of spine types).

The NTF2-like domain is essential for G3BP1 dimerization (29) and binding to another RBP, Caprin-1 (46). Therefore, inhibition of mushroom spine formation by exogenously expressing the NTF2-like domain does not necessarily infer the involvement of eIF4E. To confirm the role of eIF4E in mediating G3BP1 function, we employ the small molecule inhibitor 4EGI-1, which attenuates translation initiation by blocking the interaction between eIF4E and eIF4G (47). Since G3BP1 suppresses neuronal protein synthesis (Fig. 2), we reason that elevated protein synthesis could underlie the dendritic spine phenotype. The densities of mushroom spines and filopodia after transfection of the control shRNA were comparable to that of vector control (Fig. S4*A*). In the presence of DMSO (as vehicle control), transfection of neurons with G3BP1-shRNA resulted in the reduction of mushroom spines and the over-production of filopodia, which is consistent with our previous findings (27). However, the presence of 4EGI-1 reversed the spine defects caused by G3BP1 depletion by partially restoring the mushroom spines and completely abolishing the increase in filopodia (Fig. 4, *A* and *B*). Taken together, our findings suggest that G3BP1 promotes dendritic spine maturation by inhibiting mRNA translation, which can be achieved, at least in part, by binding to eIF4E *via* its NTF2-like domain.

### PRMT8 regulates protein synthesis and dendritic spine maturation through arginine methylation of G3BP1

**Figure 5:**
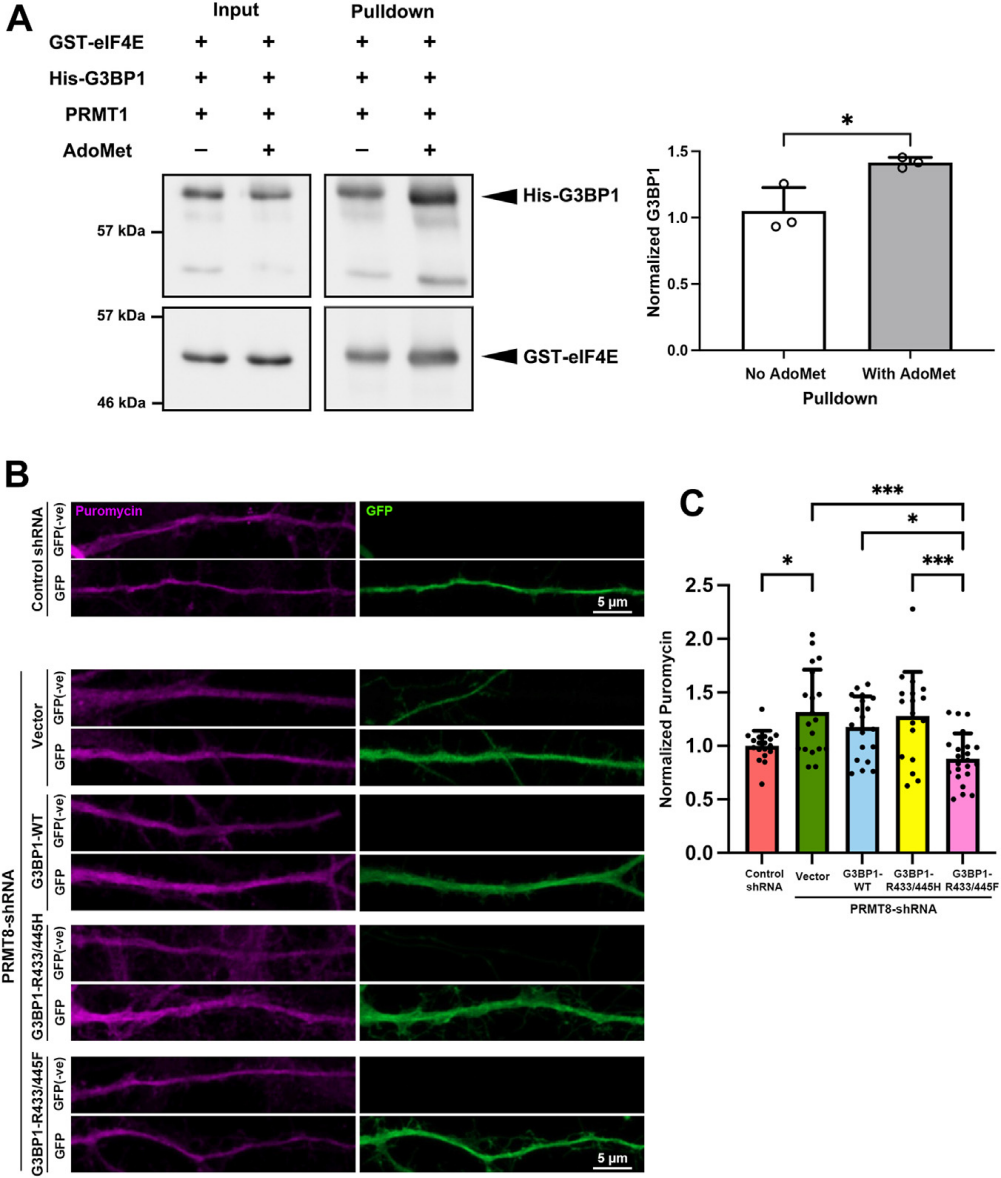
**Arginine methylation regulates the G3BP1-eIF4E interaction and protein synthesis.***A*, *in vitro* methylation of His-G3BP1 by PRMT1 increased its binding to eIF4E. Quantification showed that an increased amount of His-G3BP1 was pulled down by GST-eIF4E in the presence of the methyltransferase PRMT1 and the methyl donor AdoMet. Results were pooled from three independent experiments. Data were mean ± SD; ∗*p* < 0.05, Student’s *t* test. *B*, hippocampal neurons were co-transfected with GFP and the control- or PRMT8-shRNA with vector or different RNAi-resistant G3BP1 constructs at 13 DIV, followed by puromycin labeling at 16 DIV with GFP (*green*) staining. Representative images of dendrites of transfected neurons showing puromycin staining (*magenta*). GFP(-ve) represented dendrites from neighboring non-transfected control neurons. *C*, normalized puromycin was calculated as the ratio of fluorescence intensity from dendrites of transfected (GFP-positive, “GFP”) to the neighboring non-transfected (GFP-negative, “GFP -ve”) neurons. The ratio of the control-shRNA group was set as “1”. Results were pooled from three independent experiments; 18 to 22 dendrites from 18 to 22 neurons were quantified for each condition. Data were mean ± SD; ∗*p* < 0.05, ∗∗∗*p* < 0.001; Kruskal-Wallis test, Dunn’s multiple comparison test.

The interaction between G3BP1 and eIF4E is regulated by the RNA-binding domain (Fig. 3), which contains the C-terminal RGG motif where two arginine sites (R433 and R445) are located. We, therefore, address whether arginine methylation might regulate the G3BP1-eIF4E interaction. Toward this end, *in vitro* methylation of purified G3BP1 was induced by incubation with recombinant PRMT1, which has strong arginine methyltransferase activity on G3BP1 *in vitro* (48), before the pull-down assay. In the presence of PRMT1 and the methyl donor AdoMet, the amount of recombinant G3BP1 that was immunoprecipitated by eIF4E *in vitro* was increased, indicating that the interaction between eIF4E and G3BP1 is enhanced when G3BP1 is arginine methylated (Fig. 5*A*).

**Figure 6:**
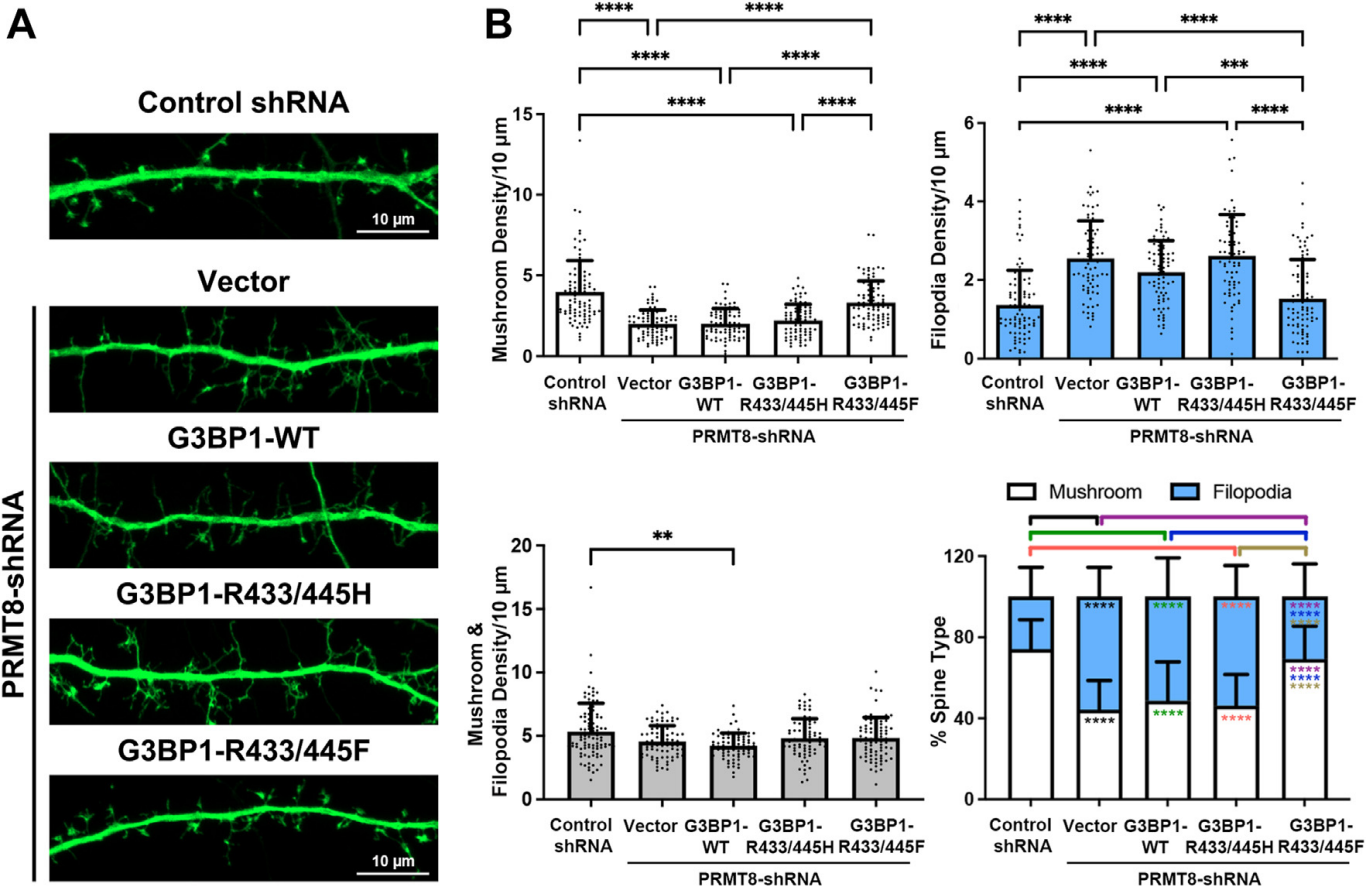
**PRMT8 regulates dendritic spine maturation through arginine methylation of G3BP1.***A*, cultured hippocampal neurons (13 DIV) were co-transfected with GFP, control-shRNA, or PRMT8-shRNA and different RNAi-resistant G3BP1 constructs, followed by GFP staining at 16 DIV. Representative images were shown. *B*, quantification indicated that only co-expression of the methylation-mimetic G3BP1 (R433/445F) but not the wild-type (WT) G3BP1 nor the methylation-deficient (R433/445H) G3BP1 rescued the decreased mushroom density and increased filopodia density caused by the PRMT8-shRNA. Results were pooled from three independent experiments; 77 to 93 dendrites from 38 to 45 neurons were quantified for each condition. Data were mean ± SD; ∗∗*p* ˂ 0.01, ∗∗∗*p* ˂ 0.001, ∗∗∗∗*p* ˂ 0.0001; Kruskal-Wallis test, Dunn’s multiple comparison test.

In non-neuronal cells, G3BP1 function is regulated by arginine methylation during the disassembly of stress granules (48, 49) and downstream of Wnt signaling (45). G3BP1 is identified as the substrate of PRMT8 in the brain (27). To conclusively address whether the function of G3BP1 in regulating protein synthesis and spine maturation depends on PRMT8-mediated arginine methylation, it is necessary to perform rescue experiments using methylation-mimetic G3BP1, in which the critical arginine sites are substituted to phenylalanine, as described for other methyltransferase substrates (50, 51, 52). If PRMT8 acts upstream of G3BP1 in neurons to coordinate mRNA translation for dendritic spine maturation, we anticipate that the defects in protein synthesis and spine morphogenesis after PRMT8 depletion can be reversed by methylation-mimetic G3BP1. We introduced the shRNA that specifically targeted PRMT8 (27) into primary hippocampal neurons and performed SUnSET assays. Like the empty vector (pSUPER), the puromycin intensity of dendrites from neurons transfected with a control shRNA was similar to that of neighboring non-transfected neurons (Fig. S4*B*). Consistent with our hypothesis, the introduction of the PRMT8-shRNA increased mRNA translation, as indicated by elevated puromycin incorporation on dendrites in the SUnSET assay, suggesting that PRMT8 mimics the role of G3BP1 in suppressing mRNA translation. Notably, co-expression of methylation-mimetic G3BP1 (R433/445F), but not the wild-type nor methylation-deficient G3BP1 (R433/445H), reversed the elevated protein synthesis caused by PRMT8 knockdown (Fig. 5, *B* and *C*). To ask whether arginine methylation of G3BP1 acts downstream of PRMT8 in regulating dendritic spine maturation, we co-expressed the different constructs of G3BP1 with PRMT8-shRNA and determine the effects on the production of mushroom spines and filopodia. Compared to control shRNA, knockdown of PRMT8 induced filopodia formation and loss of mushroom spines. Notably, co-expression of the methylation-mimetic G3BP1 could reverse the spine phenotypes. On the other hand, co-expressing either the wild-type or methylation-deficient G3BP1 failed to rescue the overproduced filopodia and reduction of mushroom spines (Fig. 6, *A* and *B*). Collectively, our results indicate that G3BP1 is the major downstream target of PRMT8 in the regulation of protein synthesis, and the methylation of G3BP1 by PRMT8 within the RGG motif is crucial for the proper maturation of dendritic spines in neurons.

## Discussions

Besides its established function in stress granule assembly, G3BP1 has been implicated to regulate different aspects of RNA metabolism, including translation, transport, stability, and decay. Additional functions of G3BP1 other than RNA metabolism have also been suggested, such as ribosomal stability and proteasome-mediated protein turnover (38). Most of these findings are derived from studying dividing cells, in which the demand for protein homeostasis is likely different from that of neurons. Moreover, a reduction of G3BP1 expression is observed in neurodegeneration (36), while G3BP1 knockout mice display selective death of neurons at birth despite being ubiquitously expressed (53). Therefore, G3BP1 likely has specific roles in the brain under physiological context, and it is crucial to elucidate the mechanism by which G3BP1 exerts its function in neurons. Here we provide multiple lines of evidence to indicate that the translation initiation factor eIF4E and the control of protein synthesis underlie the function of G3BP1 in the development of dendritic spines. eIF4E is co-immunoprecipitated with G3BP1 in the brain, and the two proteins co-localize in the dendrites of hippocampal neurons. Metabolic labeling by SUnSET assay reveals that the over-expression of G3BP1 decreases protein synthesis in neurons. Notably, exogenous expression of the NTF2-like domain of G3BP1 induces loss of mushroom spines; whereas inactivating eIF4E and translation initiation by 4EGI-1 rescues the dendritic spine defects caused by G3BP1 depletion. Other translation initiation factors in the pre-initiation complex, such as eIF3H and eIF4G, are known components of the stress granules, and they only interact with G3BP1 after induction by external stresses (40, 54). However, our present study is the first demonstration that G3BP1 binds to eIF4E without exogenous stress stimulus, and more importantly, this interaction is involved in dendritic spine maturation in neurons.

G3BP1 has been shown to either promote or inhibit the translation of its target mRNAs (35, 55). In the present study, we observe G3BP1 puncta in dendrites. SUnSET assay indicates that G3BP1 acts as a suppressor of protein synthesis in hippocampal neurons. The change in protein synthesis is small (about 20%), which agrees with a previous study that reported about a 20% increase in protein synthesis *via* metabolic labeling after depleting FMRP (56). We speculate that neurons are sensitive to changes in protein synthesis, and a more substantial increase in mRNA translation may not be readily observed after modulating the upstream regulatory pathway. Our current findings that G3BP1 suppresses protein synthesis in hippocampal neurons are consistent with Sahoo *et al.* (2018), in which G3BP1 is reported to exist as small aggregates in the axons of dorsal root ganglion neurons and negatively regulate mRNA translation. The acid-rich domain of G3BP1 and its phosphorylation on Ser-149 are involved in the regulation of axonal mRNA translation (35). Here, we demonstrate that the N-terminal NTF2-like domain of G3BP1 mediates the interaction with eIF4E for dendritic spine development in hippocampal neurons. Over-expression of the NTF2-like domain, which is anticipated to disrupt the endogenous G3BP1 function, significantly reduces the density of mushroom spines, indicating the importance of the G3BP1-eIF4E interaction in spine morphogenesis. Our additional observations that the spine defect after G3BP1 depletion is reversed by inhibiting eIF4E activity through 4EGI-1, as well as the reduction of protein synthesis after G3BP1 over-expression, further support the notion that G3BP1 promotes dendritic spine development by keeping eIF4E activity and protein synthesis under control. Exogenous expression of full-length G3BP1 suppresses the formation of filopodia. Together with our findings that G3BP1 depletion by shRNA produces more filopodia [(27) and current study], it is clear that G3BP1 is a key regulator of filopodia production during spine morphogenesis. However, expressing the NTF2-like domain of G3BP1 only reduces mushroom spine density without changing the abundance of filopodia, as opposed to G3BP1 knockdown which leads to both the decrease in mushroom spines and the over-production of filopodia. This suggests that different domains of G3BP1 are coupled to distinct downstream signaling pathways that separately control the formation of mushroom spines and filopodia.

Among the numerous translation initiation factors, eIF4E is particularly relevant to synapse development and function in neurons. Besides its role in translation initiation, eIF4E directly controls the Rac-PAK signaling pathway *via* CYFIP and WAVE1, which in turn regulates actin dynamics and dendritic spine morphology through phosphorylation of the actin-depolymerization factor cofilin (57). The eIF4E-eIF4G interaction becomes elevated in *Fmr1* knockout mice, which causes hyperactive Rac-PAK signaling and over-production of filopodia (15, 58). Of note, enhanced eIF4E-eIF4G interaction and increased actin turnover are also observed in *Prmt8* knockout brains and neurons, while the overproduction of filopodia in either PRMT8 or G3BP1-depleted neurons can be reversed by PAK1 inhibitor (27). The Rac-PAK signaling is therefore the key control point of both PRMT8 and G3BP1 in neurons. In this current study, we demonstrate that arginine methylation increases the binding between G3BP and eIF4E, while the expression of methylation-mimetic G3BP1 can rescue the elevated protein synthesis and immature spine phenotypes in PRMT8-depleted neurons. Our present findings, therefore, provide a plausible explanation of how might PRMT8 be bridged to the Rac-PAK signaling to promote dendritic spine maturation. We speculate that one major function of PRMT8 is to act on G3BP1 to regulate protein synthesis in neurons *via* eIF4E. Without PRMT8-mediated methylation, G3BP1 might release its suppression on mRNA translation. This in turn could contribute to the increased filopodia formation by activating the Rac1-PAK signaling through eIF4E, similar to what has been described for neurons lacking FMRP. Although the current study focuses on the role of PRMT8-G3BP1 in the dendritic spine maturation of developing neurons, it is possible that the two proteins also work together in other cellular contexts, such as neuronal stress response. Indeed, the spinal cord motor neurons from aged *Prmt8* null mice show decreased stress tolerance (59). Whether this compromised stress protection is contributed in part by aberrant G3BP1 function in stress granule formation and/or proteostasis remains to be determined.

RGG motif is commonly present in RBPs, and its methylation may influence the interacting capacities of several cytoplasmic RBPs with their protein/RNA partners. For instance, arginine methylation of FUS regulates its interaction with the nuclear import carrier Transportin and might be a therapeutic target for familial amyotrophic lateral sclerosis (ALS) caused by *FUS* mutations (60, 61, 62). When demethylated, the RBP HuD increases the stability of its mRNA targets by forming a tighter mRNP complex and promotes the proliferation of PC12 cells (63). Arginine methylation can also modulate different properties of FMRP, such as dimerization, interaction with polyribosomes or mRNAs (64, 65) as well as the formation of RNA granules through liquid–liquid phase separation (66). Here we found that in order for the full-length G3BP1 protein to interact with eIF4E *via* its NTF2-like domain, it requires the presence of the RNA-binding domain, in which methylation within its RGG motif further enhances the interaction. It is presently unclear how the N-terminal NTF2-like domain and the C-terminal RGG motif cooperate within the same G3BP1 molecule. However, similar intra-molecular interaction has been observed for FMRP, in which methylation of the C-terminal RGG motif affects the KH1 domain far apart to increase its dimerization with FXR1P (64). It is tempting to speculate that arginine methylation can similarly modulate other dendritically localized RBPs besides G3BP1. An important question that remains to be addressed is whether arginine methylation in neurons is regulated by external stimuli such as growth factor and synaptic activity. In this regard, it is noteworthy that methylation of the voltage-gated sodium channel Nav1.2, which is a PRMT8 substrate, is up-regulated in the brain after seizure (67). It would be interesting to explore in the future how synaptic stimuli regulates PRMT8 expression or activity locally in dendrites to modulate the methylation and function of its downstream targets.

## Experimental procedures

### DNA constructs

To knock down PRMT8, G3BP1 or eIF4E, the 19-nucleotides shRNA derived from rat PRMT8 (5′-GACTACCTCACTGTTCGAA-3′), rat G3BP1 (5′-CCTGTGTCCGACATTCAAG-3′) or rat eIF4E (5′-GAGCGGCTCCACCACTAAA-3′) nucleotide sequences were selected by the online siRNA design program (http://sirna.wi.mit.edu) and were used to create the shRNA after subcloning into the pSUPER vector. The control-shRNA sequence was 5′-GGCTACCTCCATTTAGTGT-3′. For making FLAG-tagged G3BP1, a primer was designed that contained a FLAG tag in-frame with G3BP1 at the N-terminal, and the insert was amplified by PCR from rat hippocampus cDNA and subcloned into the pcDNA3 vector at the BamHI (NEB) and NotI (NEB) restriction enzyme sites. For the construction of individual and combination of G3BP1domains tagged with FLAG, PCR was performed using the full-length rat G3BP1 construct as a template with the primers designed according to the following coding sequences of the rat G3BP1 gene: NTF2 (1–420 bp), NTF2+AcidRich+PxxP (1–1020 bp), RRM + RGG (1021–1395 bp), AcidRich+PxxP (421–1020 bp). To make RNAi-resistant constructs and different variants of G3BP1 [the methylation-deficient constructs (R433/445H) and the methylation-mimetic constructs (R433/445F)], site-directed mutagenesis using PfuUltra II Fusion HS DNA Polymerase (Agilent Technologies, Inc) was performed, and the PCR products were digested by DpnI (NEB) at 37 °C for 3 h before transformation into *E. coli* competent cells. Inserts were then subcloned into pcDNA3 vector at BamHI (NEB) and NotI (NEB) restriction enzyme sites. All expression constructs were made by PCR using the high-fidelity PfuUltra II Fusion HS DNA Polymerase. The nucleotide sequence of the insert in each plasmid was verified by Sanger sequencing.

### Primary neuronal culture

All animal experiments were approved and performed in accordance with the Animal Research Ethics Sub-Committee of City University of Hong Kong. Primary hippocampal neurons were dissociated from day 18 embryos of Sprague Dawley rats. Hippocampal neurons were cultured in a 12-well dish on 18-mm coverslips coated with poly-D-lysine (1 mg/ml, Sigma) at high density (1.4 × 10^5^ cells/18 mm coverslip) for the analysis of dendritic spines or low density (0.4 × 10^5^ cells/18 mm coverslip) for immunofluorescence staining. The hippocampal neurons were grown at 37 °C, 5% CO_2_ with Neurobasal medium (Gibco) supplemented with 2% B27 and 0.25% L-glutamine (Invitrogen).

### Transfection of primary neurons

Hippocampal neurons were transfected with different plasmids using calcium phosphate precipitation as previously described (68) with some modifications: neurons at 12 to 13 DIV were starved with prewarmed DMEM medium (Gibco, 11960044) for 2 h at 37 °C in 10% CO_2_ incubator. The prepared DNA/CaCl_2_ mixture was dropped onto cells and incubated for 13 min at 37 °C, 5% CO_2_ incubator. Hippocampal neurons were then washed with prewarmed DMEM medium and incubated for 15 min at 37 °C incubator with 10% CO_2_. Finally, neurons were transferred back to the pre-conditioned medium and incubated for 2 h in 5% CO_2_, 37 °C incubator, after which half of the medium was changed with fresh medium. Neurons were used for experiments 3 to 5 days post-transfection. The total amount of plasmids for transfection was 5 μg per coverslip. For the co-transfection experiments that involved GFP together with either the G3BP1 expression construct or shRNA, the construct of interest (2 μg) was in four-fold excess with that of GFP (0.5 μg) in the transfection mix, such that the GFP expression was a reliable indication of neurons taking up the shRNA or cDNA constructs (Fig. S3).

### Western blot analysis

Cells or tissues were collected with cold 1X RIPA lysis buffer (0.5% sodium deoxycholate, 1% NP-40, 0.1% SDS in D-PBS) plus protease and phosphatase inhibitors (10 μg/ml soybean trypsin inhibitor, 10 μg/ml leupeptin, 10 μg/ml aprotinin, 2 μg/ml antipain, 30 nM okadaic acid, 5 mM benzamidine, 1 mM sodium orthovanadate, 1 mM PMSF, 1 mM sodium fluoride, 100 mM beta-glycerophosphate). After centrifuging at 4 °C, 13,000 rpm for 10 min, the supernatant was collected. 1X sample buffer (5X sample buffer: 300 mM Tris-HCl buffer, pH 6.8, 10% (w/v) SDS, 25% (v/v) beta-mercaptoethanol, 50% (v/v) glycerol, 0.05% (w/v) bromophenol blue) was added to protein extract which was boiled at 100 °C for 5 min with heat block. Equal amounts of protein samples were subject to SDS-PAGE for separation and transferred onto PVDF membranes (Pall). The membranes were blocked with blocking buffer (5% non-fat milk in TBST) for 1 h at room temperature, followed by incubation with primary antibody (diluted with 5% BSA and 0.02% sodium azide in TBST) overnight at 4 °C. The following antibodies were used at the indicated dilutions: G3BP1 (1:2000, Bethyl A302-033A), eIF4E [1:1000, Cell Signaling Technology #9742S; the band denoted as eIF4E (1) in Fig. 1*A*)], eIF4E [1:1000, Bethyl A301-154A; the band denoted as eIF4E (2) in Fig. 1*A*)], FLAG (1:3000, Sigma-Aldrich #F1804)]. After washing with TBST, the membranes were incubated for 1 h at room temperature with HRP-conjugated secondary antibody diluted 1:3000 in 5% non-fat milk in TBST. The following secondary antibodies were used: anti-mouse IgG, HRP-linked antibody (Cell Signaling Technology #7076S), and anti-rabbit IgG, HRP-linked antibody (Cell Signaling Technology #7074S). The HRP signal was detected by the SuperSignal West Pico PLUS Chemiluminescent Substrate (Thermo Fisher Scientific) using the ChemiDoc MP imaging system (BioRAD).

### Immunoprecipitation and pull-down assay

HEK-293T cells were cultured in DMEM medium (Gibco) plus 10% FBS and 1% penicillin-streptomycin in 5% CO_2_, 37 °C incubator. Cells were grown to 70 to 85% confluence and were transfected with Lipofectamine LTX reagent and plus reagent (Invitrogen) in accordance with the manufacturer’s protocols. 24 h post-transfection, cell lysate was collected using either cold 1X RIPA lysis buffer containing various protease and phosphatase inhibitors for immunoprecipitation (IP), or cold 1X NP-40 lysis buffer [50 mM tris buffer (pH 8.5), 50 mM NaCl, 0.5% NP-40 in MilliQ H_2_O] containing various protease and phosphatase inhibitors for co-IP.

For the immunoprecipitation of FLAG-G3BP1, the lysate (1 mg) was incubated with FLAG beads (15 μl, Sigma) for 2 h at 4 °C to immunoprecipitate the FLAG-tagged proteins. After washing three times by lysis buffer containing the various protease and phosphatase inhibitors, proteins were eluted with 2.5X sample buffer with 100 μM DTT for Western blot analysis. For the immunoprecipitation of G3BP1, lysate (1 mg) was incubated with G3BP1 antibody (1 μg, Bethyl A302-033A) overnight at 4 °C. After incubation with Protein A beads (40 μl, GE Healthcare) at 4 °C for 1 h, the beads were washed four times with lysis buffer containing various protease and phosphatase inhibitors, and proteins were eluted with 2.5X sample buffer with 100 μM DTT for Western blot analysis.

### *In vitro* binding assay and *in vitro* methylation

To detect the *in vitro* binding between G3BP1 and eIF4E, the purified recombinant proteins His-G3BP1 (2 μg, Novus) and GST-eIF4E (4 μg, Cayman) were mixed in Tris buffer containing various proteinase inhibitors and rotated overnight at 4 °C. GSH beads (50 μl, GE HealthCare) were added to the mixture and followed by 2 h rotation at 4 °C. The beads were washed three times with Tris buffer containing proteinase inhibitors and eluted with 2.5X sample buffer with 100 μM DTT. For the *in vitro* methylation of G3BP1, the purified proteins His-G3BP1 (2 μg), GST-eIF4E (4 μg), PRMT1 (4 μg, NKMAX), and AdoMet (80 μM, Sigma) were mixed in DPBS at 30 °C for 1 h, followed by binding assay by adding the reaction mix and GSH beads (50 ul) into D-PBS with proteinase inhibitors and rotated for 2 h in 4 °C as described above. After elution, samples were boiled at 100 °C for 5 min before SDS-PAGE and Western blot analysis.

### Immunofluorescence staining

Cultured hippocampal neurons were fixed with 4% PFA and 4% sucrose solution for 15 min at room temperature. After washing, the cells were blocked with blocking buffer [0.4% Triton X-100 (v/v) and 1% BSA (v/v)] for 45 min at room temperature, followed by incubation with primary antibodies [eIF4E, Cell Signalling #9742S at 1:100, a dilution described by previous study (69)] and GFP (Invitrogen A-11120 at 1:2000) overnight at 4 °C. On the following day, the cells were washed three times with washing buffer (0.02% Triton X-100 and 0.25% BSA in D-PBS) and incubated with Alexa-conjugated secondary antibodies (Invitrogen A-21236 and A-21131) at 1:1000 in D-PBS containing 0.02% Triton X-100 and 1% BSA for 45 min at room temperature in dark. After being washed with washing buffer for two times, cells were rinsed with D-PBS and sterilized MilliQ H_2_O, and the coverslips were mounted on slides (Thermo Fisher Scientific) with hydromount medium (National Diagnostics).

To examine dendritic spine morphology, the transfected hippocampal neurons were fixed at 16 DIV and incubated overnight at 4 °C with anti-GFP antibody diluted in 1X GDB buffer (0.1% Gelatine, 0.3% TritonX, 0.45 M NaCl, 16.5 mM phosphate buffer, pH 7.4). On the following day, the cells were incubated with Alexa-488 conjugated secondary antibody for 45 min in dark at room temperature, followed by washing three times with filtered phosphate buffer (20 mM phosphate buffer and 0.5 M NaCl) and mounting.

### SUnSET assay

Cultured hippocampal neurons (16–17 DIV) were incubated with puromycin (1.8 μM) for 10 min in medium at 37 °C with 5% CO_2_. In the control experiment with protein synthesis inhibitor, cells were pre-treated with anisomycin (40 μM) for 30 min before addition of puromycin to the medium. Incubation was stopped by two fast washes in PBS (1 × PBS, pH 7.4, 1 mM MgCl_2_, 0.1 mM CaCl_2_), and cells were fixed in PFA-sucrose for 15 min. After fixation and washing three times with D-PBS, cells were permeabilized in buffer (0.5% TX in D-PBS) for 15 min and incubated with blocking buffer (3% BSA in D-PBS) for 30 min. After blocking, the cells were incubated with puromycin antibody (Kerafast #EQ0001, 1:500 diluted in blocking buffer) overnight at 4 °C and subjected to immunofluorescence staining on the following day. For the triple staining against G3BP1, puromycin, and GFP, neurons were fixed and blocked as above. They were then incubated overnight at 4 °C with the following primary antibodies: Mouse anti-Puromycin (1:500, Kerafast #EQ0001), Rabbit anti-G3BP1 (1:100, Bethyl A302-033A), and Chicken anti-GFP (1:2000, Aves Labs GFP-1020) that were diluted in blocking buffer. On the following day, the cells were washed three times with washing buffer (0.02% Triton X-100 and 0.25% BSA in D-PBS) and incubated with Alexa-488 anti-Chicken, Alexa-647 anti-Mouse, Alexa-546 anti-Rabbit conjugated secondary antibodies diluted at 1:1000 in D-PBS containing 0.02% Triton X-100 and 1% BSA for 45 min at room temperature in dark. After being washed with washing buffer for two times, cells were rinsed with D-PBS and sterilized MilliQ H_2_O, and the coverslips were mounted on slides with hydromount medium.

### Confocal imaging acquisition and analysis

Fluorescence images of dissociated hippocampal neurons were acquired using either the Carl Zeiss LSM 900 with the fast Airyscan mode (resolution: 1024 × 1024, optical zoom: 0.6–1 X, 16-bit image, averaging number:1, interval: 0.15 μm, pinhole: 1.15–1.16 AU for each channel) or the Carl Zeiss 880 microscopes (scan speed: 1.03 μs, resolution: 1024 × 1024, optical zoom: 0.6, 8-bit image, pinhole: 1 AU for each channel, and z-interval: 0.4 μm), with the 63x oil-immersion lens (N.A. 1.4). Images were exported with Zen software and quantified with MetaMorph and Image J software.

Analysis of dendritic spine morphology was performed as previously reported (27, 70). Segments of secondary apical dendrites (length: 50–70 μm) were selected blindly and cropped in Photoshop for quantification. The dimensions of the width of spine head (H), neck width (N) and total length (L) were measured using the Nikon NIS-Elements Advanced Research software. Based on these values, the dendritic spines were grouped into mushroom spines and filopodia: mushroom spines: H/N ≥1.5. Filopodia: H/N <1.2 & L/N >3. To quantify the fluorescence intensity of puromycin and G3BP1 staining, fluorescence images were acquired with identical parameters. Because of variations of staining intensities across the same sample, the puromycin staining intensities of dendrites from the transfected (GFP-positive) and neighboring non-transfected (GFP-negative) neurons within the same imaging fields were compared to calculate the changes in puromycin incorporation for the different experimental conditions. Secondary dendrites with similar width and distance from the cell body were selected from the transfected (GFP-positive) and nearby non-transfected control cells (GFP-negative) to minimize variations due to differences in dendritic volumes and relative proximity to the soma. Dendrites were outlined manually in Image J software and the average intensity in the ROI was measured by an investigator blinded to the sample identity. Normalized puromycin of each transfected neuron was then calculated as the ratio of puromycin fluorescence intensity from its dendrite to that of non-transfected control neuron. The ratio of fluorescence intensity between transfected and non-transfected neurons was set as “1” in either the GFP control group (Fig. 2*B*) or the control-shRNA group (Fig. 5*C*).

To analyse the colocalization of eIF4E and tdTomato-G3BP1, the ImageJ plug-in software “JACoP” was used to calculate both the Manders’ coefficients (M1 and M2) and Pearson’s coefficient. M1 refers to the fraction of pixels in eIF4E which overlap with pixels of tdTomato-G3BP1, whereas M2 refers to the fraction of pixels in tdTomato-G3BP1 which overlap with pixels of eIF4E. Both Manders’ coefficients range from 0 to 1, with 0 indicates no correlation and 1 indicates perfect correlation. Image thresholding using the JACoP plug-in was set manually for each image to calculate the Manders’ coefficients. Pearson’s correlation ranges from −1 to 1, with −1 represents complete exclusion of pixels of eIF4E from tdTomato-G3BP1 and vice-versa; whereas 0 indicates a random correlation and 1 signifies perfect correlation.

### Statistical analysis

All values were shown as mean ± SD and analyzed in GraphPad Prism software. Each group of values was analyzed by the Shapiro–Wilk normality test. For the groups of data that passed the Shapiro-Wilk normality test, data with two experimental groups were analyzed by Student’s *t* test, while data with more than two experimental groups were analysed with One-way ANOVA followed by Tukey post-hoc test. For the groups of data which did not obey the Shapiro–Wilk normality distribution, data with two groups were analyzed by Mann–Whitney test, and results with at least three groups were analyzed with the Kruskal-Wallis test followed by Dunn’s multiple comparison test. Statistical significance was defined as *p* <0.05.

## Data availability

The authors declare no restrictions on data availability.

## Supporting information

This article contains supporting information.

## Conflict of interest

The authors declare that they have no conflicts of interest with the contents of this article.
