# Supplementary material for: The translation initiating factor eIF4E and arginine methylation underlie G3BP1 function in dendritic spine development of neurons
Source: J Biol Chem. 2023 Jul 11;299(8):105029. doi: 10.1016/j.jbc.2023.105029 (PMC10432808; doi:10.1016/j.jbc.2023.105029)
Supplement: Supporting Figures S1–S4 [file mmc1.pdf]

Supporting Information

The translation initiating factor eIF4E and arginine methylation underlie G3BP1 function in dendritic spine development of neurons

Rui Dong[1*], Xuejun Li[1,2,*], Angelo D. Flores[1], and Kwok-On Lai [1,2#]

[1] Department of Neuroscience, [2] Hong Kong Institute for Advanced Study
City University of Hong Kong, Hong Kong

[*]Both authors contributed equally to this work
[#] To whom correspondence should be addressed:
Dr. Kwok-On Lai
Department of Neuroscience, City University of Hong Kong
1A-405, 4/F, Block 1, To Yuen Building, 31 To Yuen Street
City University of Hong Kong, Tat Chee Avenue, Kowloon, Hong Kong
Phone: 852-3442-4152
E-mail: kwokolai@cityu.edu.hk

Running title: eIF4E is a downstream target of G3BP1 in neurons

Keywords: dendritic spine, synapse, arginine methylation,
RNA-binding protein, signal transduction

Supplementary Figure 1

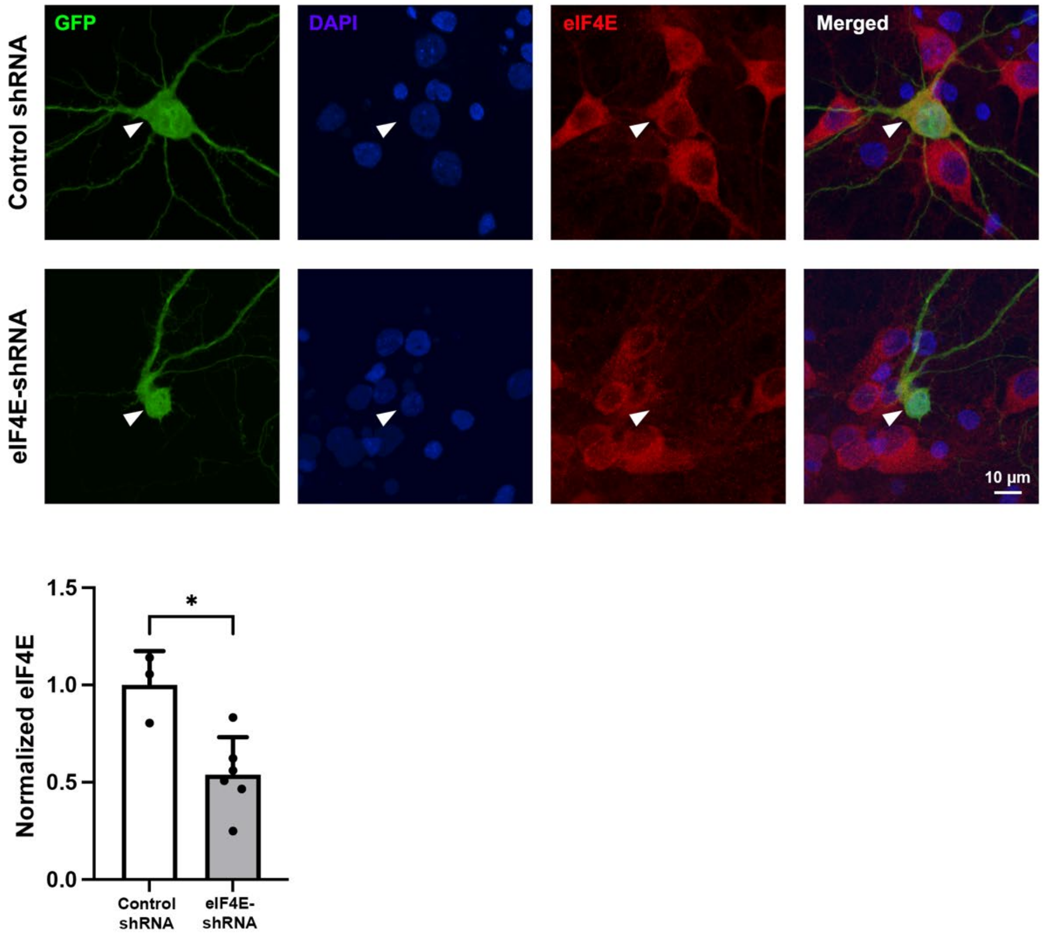

Figure S1. Testing the specificity of eIF4E antibody for immunofluorescence staining of hippocampal neurons

Hippocampal neurons were co-transfected with GFP and control shRNA or eIF4E shRNA on 12 DIV, and fixed on 16 DIV followed by staining with eIF4E antibody and DAPI. Representative images of the co-transfected GFP-positive neurons were shown (arrowheads). Compared to neurons transfected with control shRNA, the eIF4E signal (red) was significantly decreased in neurons that took up the eIF4E shRNA, indicating specificity of the antibody (3-5 neurons were analyzed from one experiment). Data were mean ± SD; *$p < 0.05$; Student's *t*-test.

Supplementary Figure 2

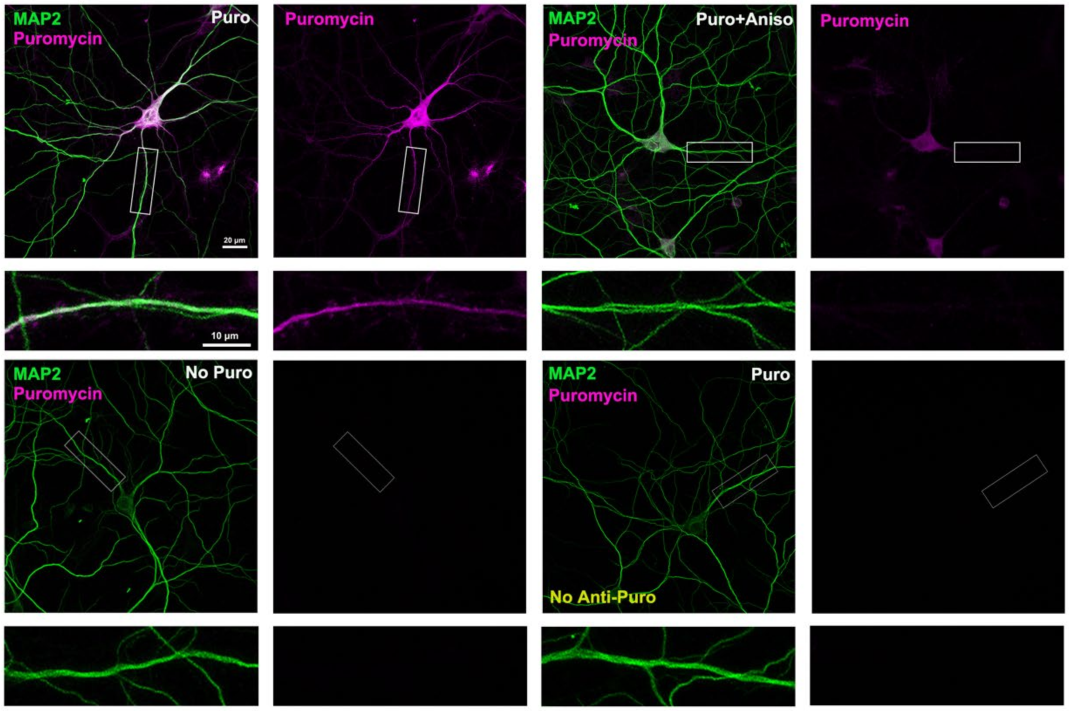

Figure S2. Determine protein synthesis in neurons *in situ* using the SUnSET assay

Images of cultured hippocampal neurons (17 DIV) stained by MAP2 antibody (green). The newly synthesized proteins (magenta) were detected after 10 min of puromycin labeling (Puro, upper left). The validity of the assay was confirmed by pre-incubation with the protein synthesis inhibitor anisomycin (Puro + Aniso, upper right), which largely reduced the puromycin signals. Additional negative controls include eliminating puromycin during the labeling period (No puro, lower left) or the absence of puromycin antibody (No Anti-Puro, lower right).

Supplementary Figure 3

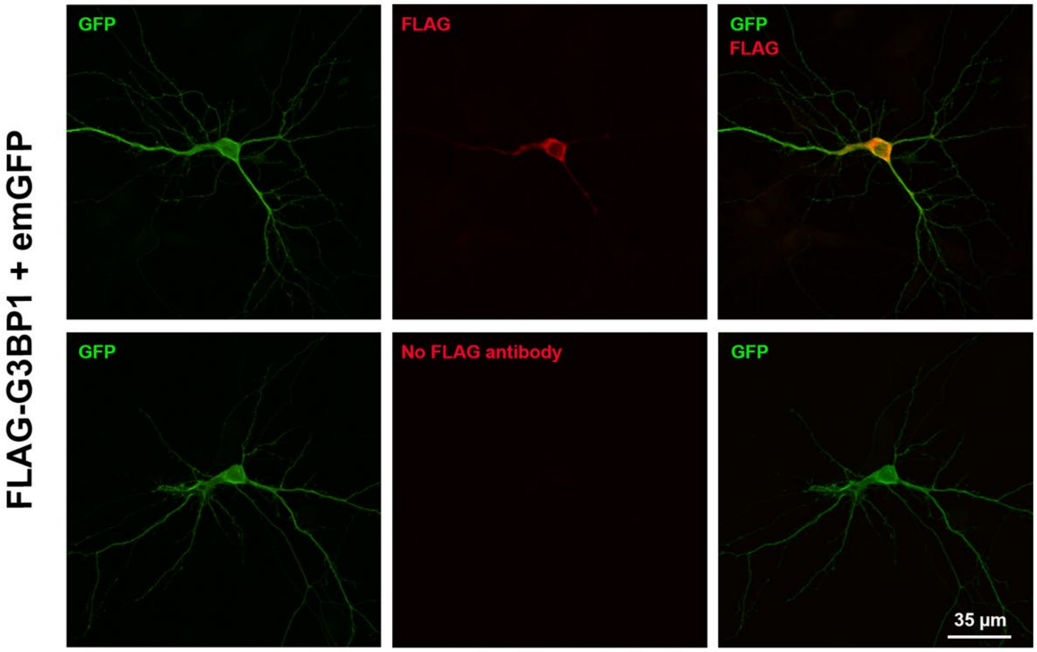

Figure S3. GFP signal indicates the presence of co-transfected plasmid in cultured hippocampal neurons

Hippocampal neurons were transfected with GFP and FLAG-G3BP1 at a ratio of 1 to 4 at 12 DIV, followed by staining with GFP (green) and FLAG (red) antibodies at 16 DIV. Representative images of the co-transfected neurons were shown. No FLAG antibody (lower panel) acted as negative control to eliminate the crosstalk of the two imaging channels. All the randomly selected GFP-positive neurons exhibited FLAG immunoreactivity, indicating that under this ratio of plasmids GFP is a reliable indicator for the presence of the co-transfected plasmid (37 neurons from one experiment were imaged).

Supplementary Figure 4

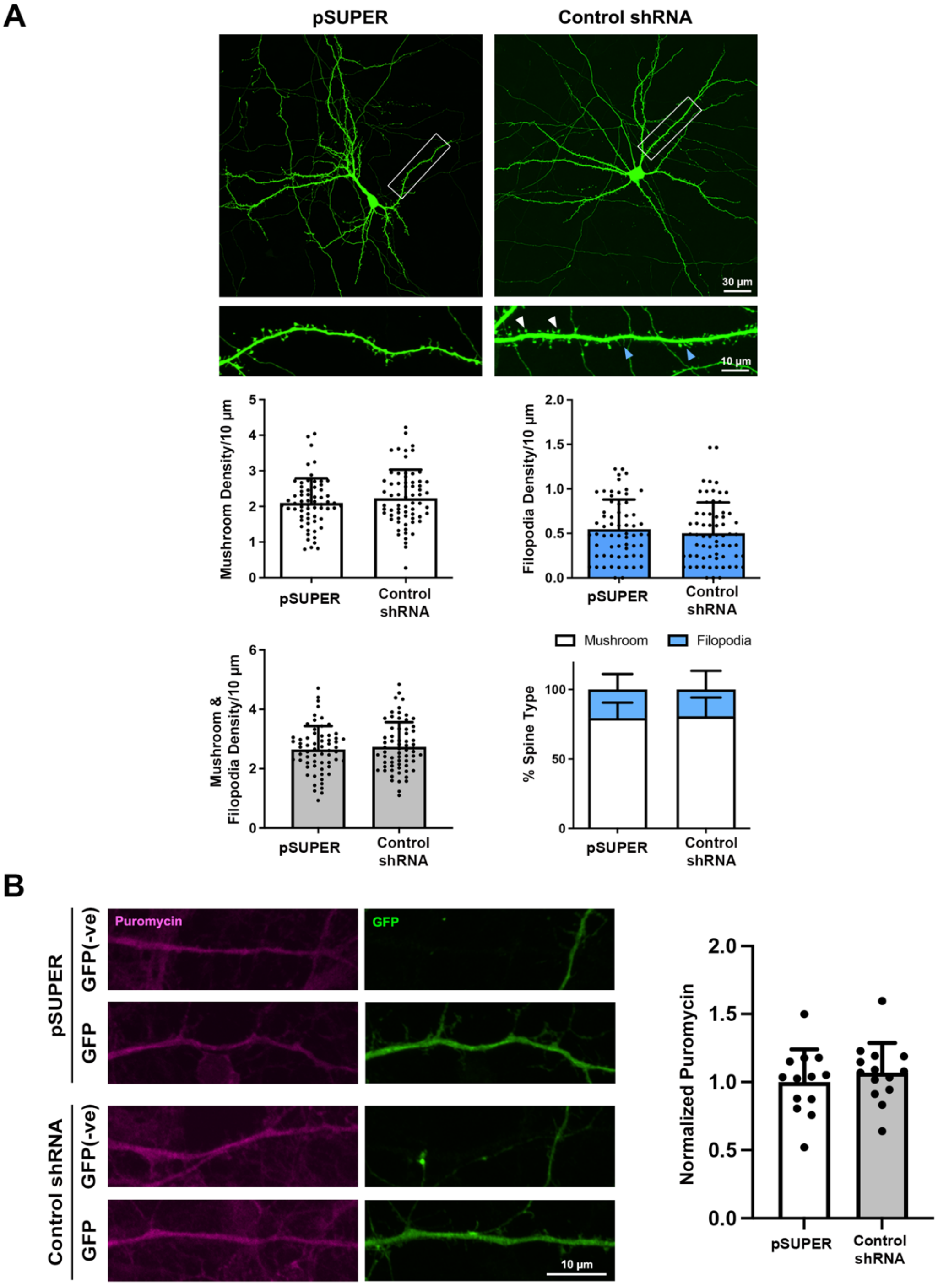

Figure S4. Control shRNA is innocuous in dendritic spine development and protein synthesis of neurons

A. Representative images showing the neurons co-transfected with GFP and either pSUPER vector or control shRNA on 13 DIV, followed by staining of GFP on 16 DIV. The density and percentage of mushroom spines and filopodia, as well as their relative proportions were similar between the control shRNA and the vector control. Mushroom spines and filopodia were indicated by white and blue arrowheads, respectively. Results were pooled from two independent experiments; 66-68 dendrites from 22-23 neurons were quantified for each condition. Data were mean ± SD; Student's *t*-test.

B. Representative images showing the neurons co-transfected with GFP and either pSUPER vector or control shRNA on 12 DIV, followed by SUnSET assay on 16 DIV. There was no significant difference in puromycin signal between the vector control and the control shRNA (results pooled from two different experiments; 13-14 dendrites from 13-14 cells were analyzed). Data were mean ± SD; Student's *t*-test.
